# Supplementary material for: Analyses of the Global Multilocus Genotypes of the Human Pathogenic Yeast Cryptococcus neoformans Species Complex
Source: Genes (Basel). 2022 Nov 6;13(11):2045. doi: 10.3390/genes13112045 (PMC9691084; doi:10.3390/genes13112045)
Supplement: Supplementary file 1 [file genes-13-02045-s001.zip › Supplementary Figure S1-S7 - Phylogenic tree relationships among genes of CNSC.pdf]

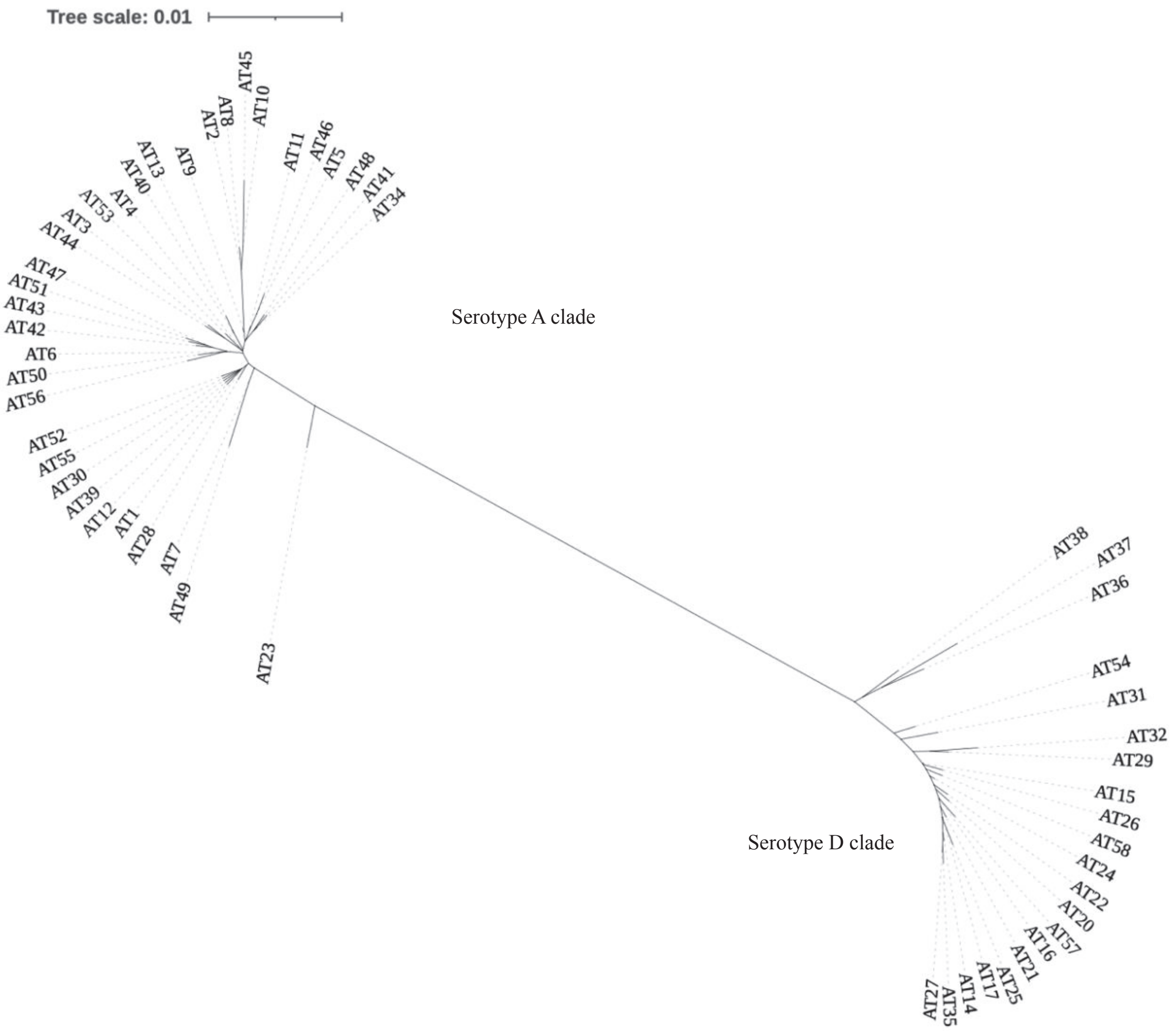

Supplement Figure S1. Phylogenic tree showing relationships among 55 allele types (AT) of the *CAP59* gene

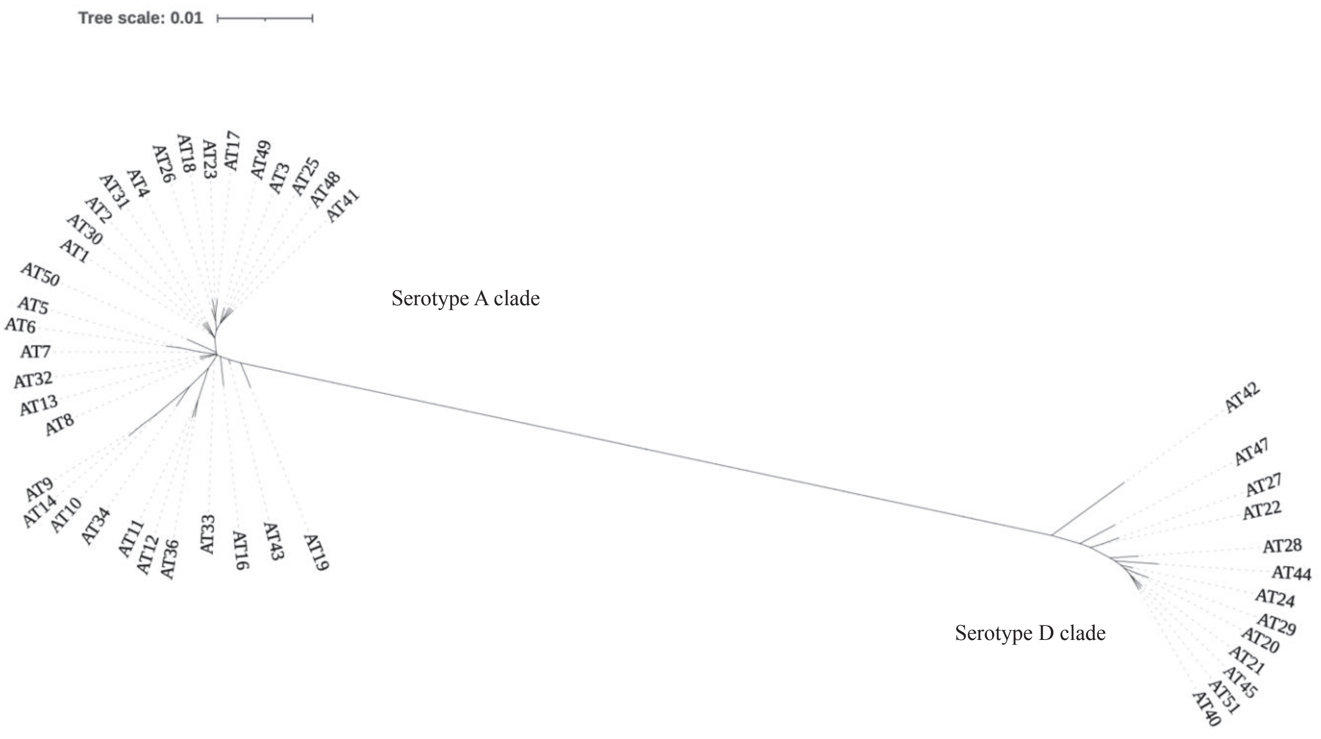

Supplement Figure S2. Phylogenic tree showing relationships among 45 allele types (AT) of the *GPD1* gene

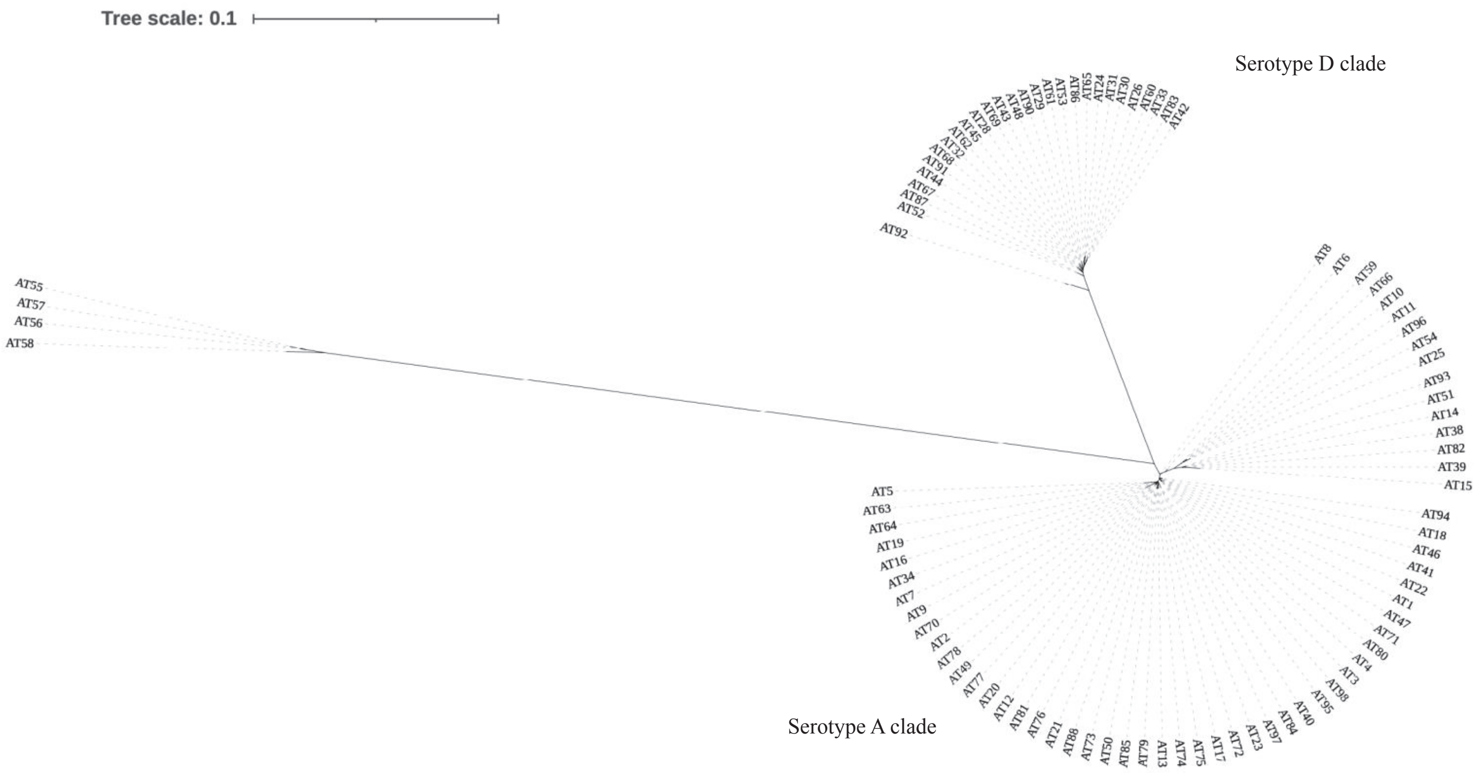

Supplement Figure S3. Phylogenic tree showing relationships among 93 allele types (AT) of the *IGS1* gene

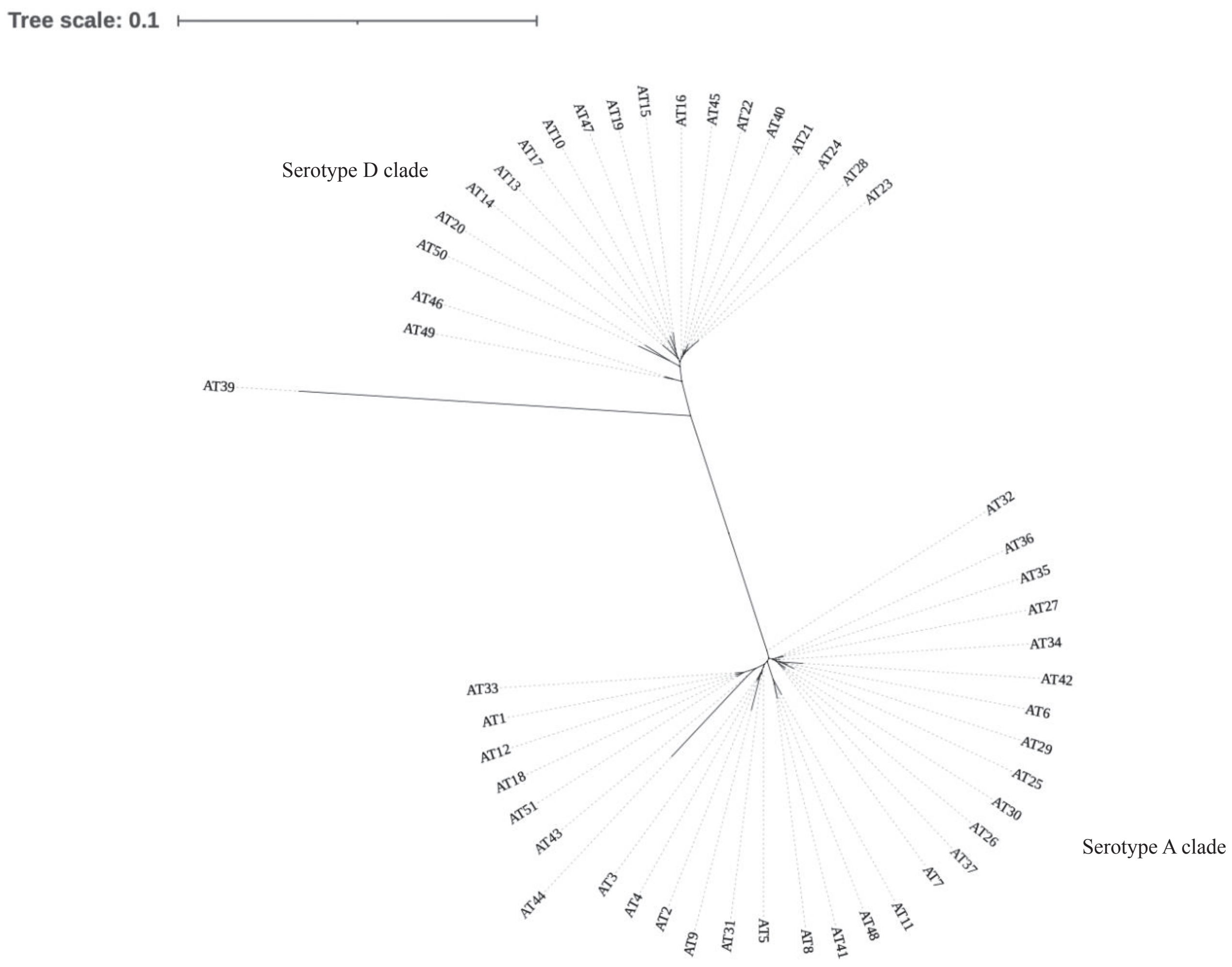

Supplement Figure S4. Phylogenic tree showing relationships among 50 allele types (AT) of the *LAC1* gene

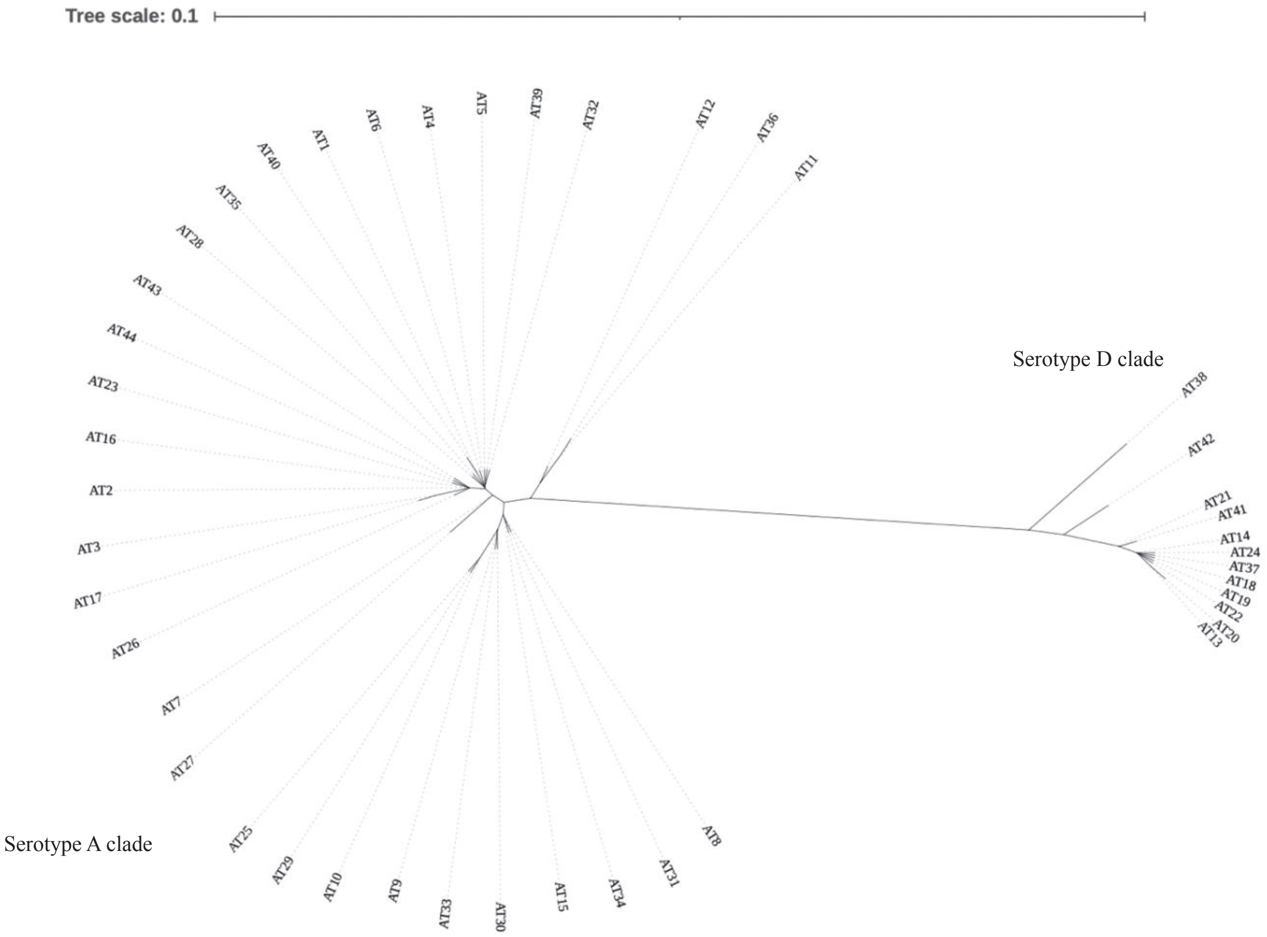

Supplement Figure S5. Phylogenic tree showing relationships among 44 allele types (AT) of the *PLB1* gene

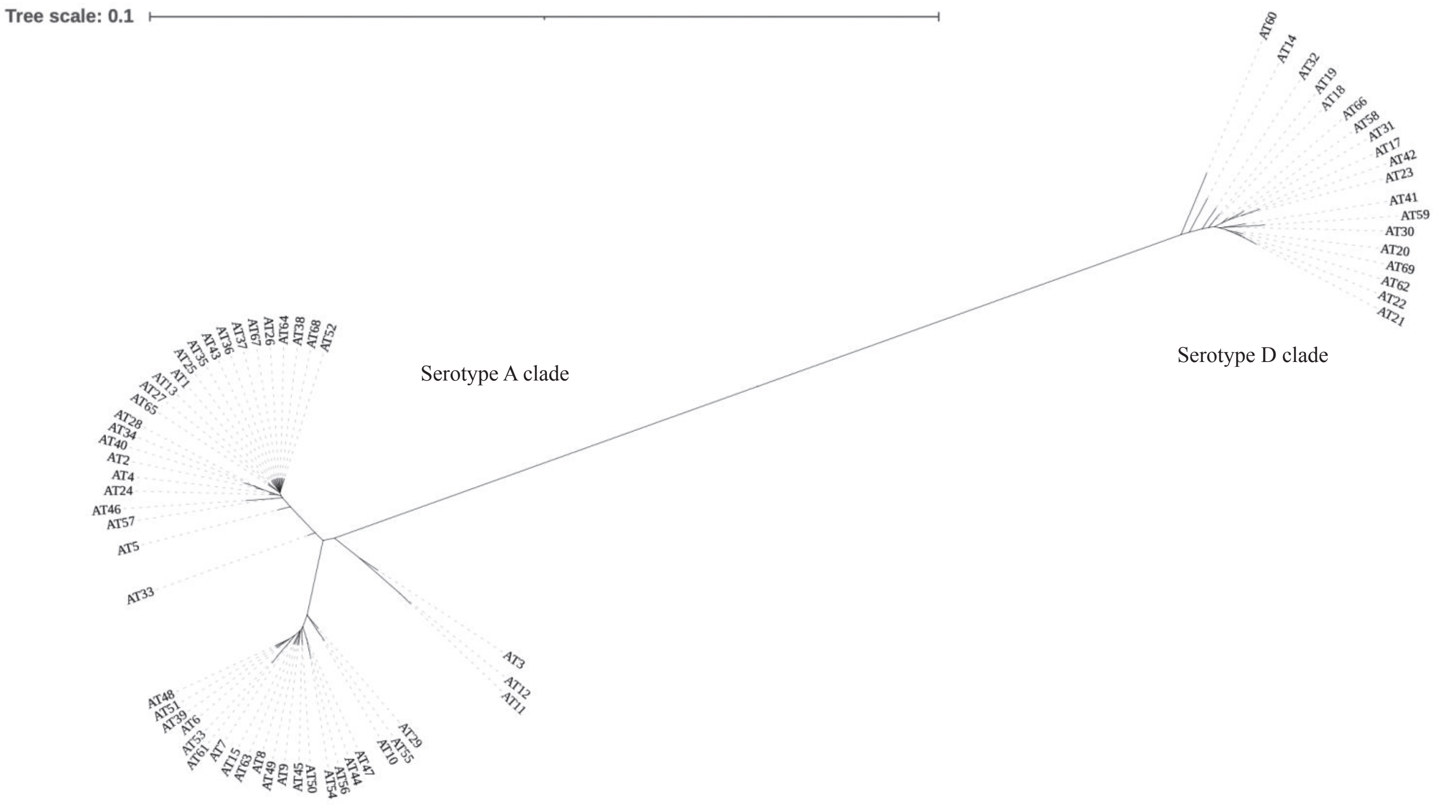

Supplement Figure S6. Phylogenic tree showing relationships among 68 allele types (AT) of the *SOD1* gene

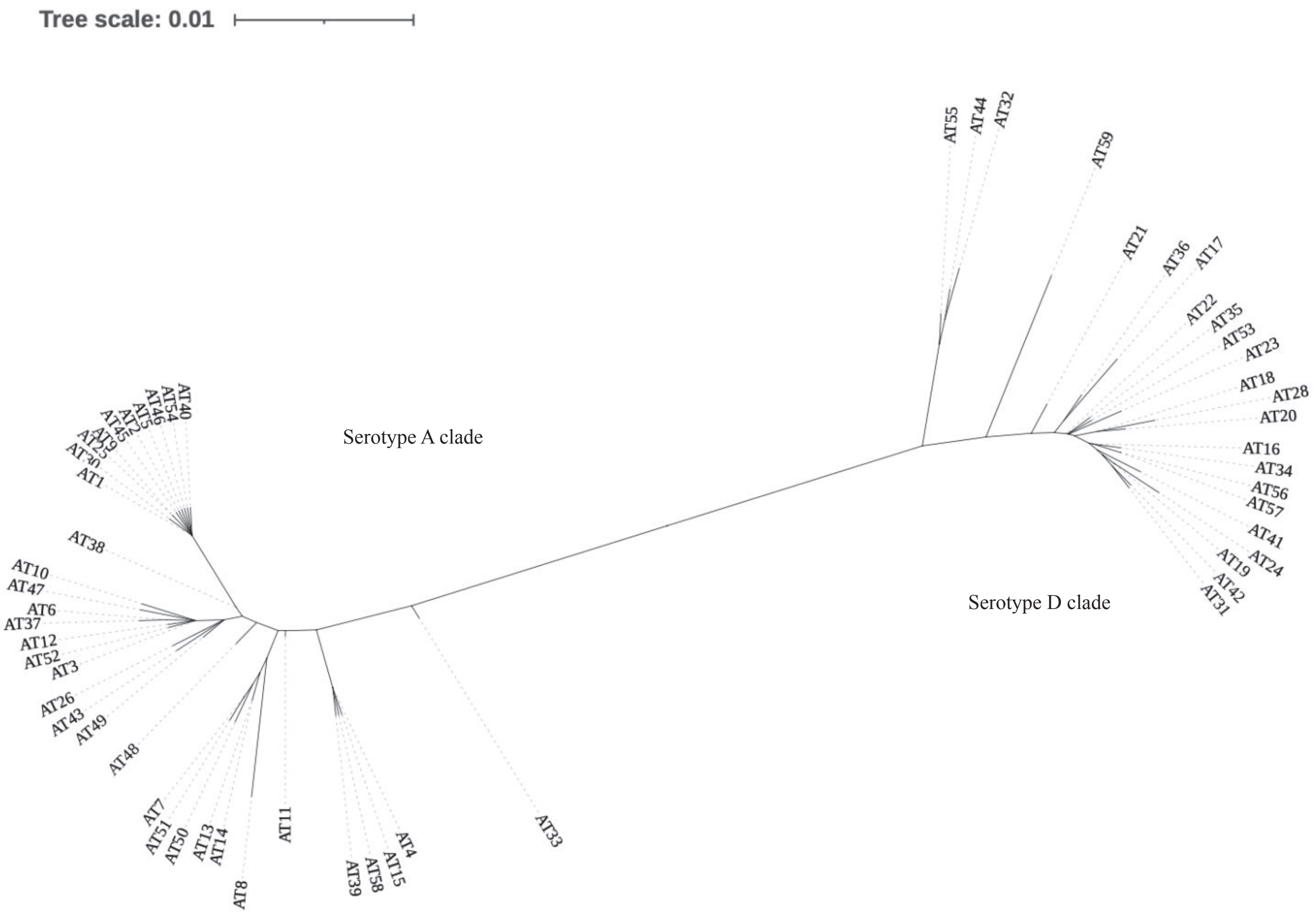

Supplement Figure S7. Phylogenic tree showing relationships among 57 allele types (AT) of the *URA5* gene
